# Supplementary material for: Public acceptance of privacy-encroaching policies to address the COVID-19 pandemic in the United Kingdom
Source: PLoS One. 2021 Jan 22;16(1):e0245740. doi: 10.1371/journal.pone.0245740 (PMC7822290; doi:10.1371/journal.pone.0245740)
Supplement: Attachment — Submitted filename: R2RPONE.pdf [file pone.0245740.s001.pdf]

PONE-D-20-26680

Public acceptance of Privacy-Encroaching Policies to Address the COVID-19 Pandemic in the United Kingdom

by

Lewandowsky et al.

Response to reviewers

(Original text in `courier font`, replies in *black italics.*)

From the action letter

1. Please ensure that your manuscript meets PLOS ONE's style requirements, including those for file naming. The PLOS ONE style templates can be found at

https://journals.plos.org/plosone/s/file?id=wjVg/PLOSOne_formatting_sample_main_body.pdf and

https://journals.plos.org/plosone/s/file?id=ba62/PLOSOne_formatting_sample_title_authors_affiliations.pdf

In the interest of reproducibility and transparency, our paper is produced by R Markdown and combines the R code for the analysis and all text. Each compilation of the paper produces the results and figures from scratch, thereby guarding against transcription errors and other glitches when changes are made to the analysis.

We have used the PLOS ONE LaTeX template in conjunction with a Markdown script available online to generate this version. Given that we used a template provided by the publisher, we expect our manuscript to conform to the required style.

2. Please provide additional details regarding participant consent. In the ethics statement in the Methods and online submission information, please ensure that you have specified what type you obtained (for instance, written or verbal, and if verbal, how it was documented and witnessed). If your study included minors, state whether you obtained consent from parents or guardians. If the need for consent was waived by the ethics committee, please include this information.

We have provided the additional information in the Method section.

Reviewers' Comments to the Author

1. Is the manuscript technically sound, and do the data support the conclusions?

The manuscript must describe a technically sound piece of scientific research with data that supports the conclusions. Experiments must

have been conducted rigorously, with appropriate controls, replication, and sample sizes. The conclusions must be drawn appropriately based on the data presented.

Reviewer #1: Yes

Reviewer #2: Partly

---

2. Has the statistical analysis been performed appropriately and rigorously?

Reviewer #1: Yes

Reviewer #2: Yes

---

3. Have the authors made all data underlying the findings in their manuscript fully available?

The PLOS Data policy requires authors to make all data underlying the findings described in their manuscript fully available without restriction, with rare exception (please refer to the Data Availability Statement in the manuscript PDF file). The data should be provided as part of the manuscript or its supporting information, or deposited to a public repository. For example, in addition to summary statistics, the data points behind means, medians and variance measures should be available. If there are restrictions on publicly sharing data—e.g. participant privacy or use of data from a third party—those must be specified.

Reviewer #1: Yes

Reviewer #2: Yes

---

4. Is the manuscript presented in an intelligible fashion and written in standard English?

PLOS ONE does not copyedit accepted manuscripts, so the language in submitted articles must be clear, correct, and unambiguous. Any typographical or grammatical errors should be corrected at revision, so please note any specific errors here.

Reviewer #1: Yes

Reviewer #2: Yes

---

5. Review Comments to the Author

Please use the space provided to explain your answers to the questions above. You may also include additional comments for the author, including concerns about dual publication, research ethics,

or publication ethics. (Please upload your review as an attachment if it exceeds 20,000 characters)

Reviewer #1: There are items for further explanations or clarifications:

1. The first survey was conducted on 28 and 29 March, and the second on 16 April. This means that the interval between the two is less than 3 weeks. If there is a reason for this short interval, it should be explained. Between the two surveys, perhaps the only significant difference explained in the article is the number of those who were infected (and died). If so, it would have been easily expected that the results from the two surveys would not be significantly different. The actual results indeed are not very different between the two.

That is correct, the results did not differ much. That said, given that the deaths increased 13-fold during that relatively brief period, one might have expected larger differences than we found.

2. Statistical discrepancies depending on scenarios (Mild, Severe, and Bluetooth) are not very large. One possible reason for this could be the lack of understanding about the differences among the scenarios. For instance, the Mild scenario is presumably a centralized scheme, whereas the Bluetooth scenario is a de-centralized scheme. Survey participants may or may not have fully appreciated the nuanced social and technological differences associated with each of the scenarios. The extent to which the survey participants may or may not have understood these differences need to be explained more clearly.

This is an interesting possibility. However, the items in Table 2, which query the potential harm from the app, should put those concerns to rest. As shown in Figure 6 (bottom panel), people are very aware of how much control they have over their data in the different scenarios. Virtually all participants recognize, for example, that the severe scenario offers very little control. We therefore believe that our participants understood the differences between scenarios. We now highlight this in the paper.

Reviewer #2: In the paper “Public acceptance of Privacy-Encroaching Policies to Address the COVID-19 Pandemic in the United Kingdom,” the authors conducted two surveys with participants from the United Kingdom. The first survey was conducted during the first wave of COVID-19, and the second survey during the second wave. Results are presented and discussed.

I think it’s important that you explain the privacy paradox and privacy calculus early in the paper, which have become important aspects in privacy research. However, later this is only discussed at a surface level. Please discuss much more in-depth. What are the implications for the privacy paradox and privacy calculus?

We have expanded the discussion of the privacy-related issues.

Please outline early in the paper what the main objective and contribution of the paper is, both for research and practice. In the last paragraph of the section "Immunity passports" you describe what you are doing in the paper but the objective and contribution is not clear. Please move this paragraph to the end of the introduction.

We have rewritten the Introduction to make our objectives clearer. In particular, we have inserted a paragraph early on that summarizes our objectives before we turn to the details of what motivated our particular research.

The method is described well, however, you could improve on transparency. In the section "Overview" you mention that an app was used. Did you develop the app? Is it a third-party app? Which app is it actually? Please provide links and screenshots. Did participants actually used the app?

There was no app, as explained on p. 7 (original version). However, we now clarify this further by making it even more explicit that this was a survey that probed people's responses to hypothetical scenarios that describe an app. It must be recalled that this survey was conducted at a time when no such app was actually available for download in the U.K. (as we state in the Method section).

Please describe in detail how the items were developed. Are they self-developed or did you draw on items from other prior studies? If self-developed, describe in detail the process of developing the items.

We describe this in the Method section.

The results are interesting but it's not clear how it will help for the current and any future pandemics. Is the main message that governments should build trust in government and reduce perceived harms of tracking policy? It appears to me that this is a too simplistic approach.

This is indeed one aspect of our conclusion. There are additional aspects that we consider in greater depth in the revision Discussion, such as the importance of knowing how long the data would be stored and the somewhat surprising finding that there was relatively little difference in endorsement between the mild and severe scenarios.

Important literature is missing. Please see the following review articles for reference to further literature:

We thank the reviewer for the pointers to additional literature which we have read with great interest. We cite the relevant papers in the revision.

Belanger, F., Crossler, R. E. 2011. Privacy in the Digital Age: A Review of Information Privacy Research in Information Systems. MIS Quarterly, 35(4), pp. 1017-1041. https://doi.org/10.2307/41409971

Smith, H. J., Dinev, T., Xu, H. 2011. Information Privacy Research: An Interdisciplinary Review. MIS Quarterly, 35(4), pp. 989-1015.

https://doi.org/10.2307/41409970

You might also want to have a closer look at privacy literature with a specific mobile app context, see for example:

Degirmenci, K. 2020. Mobile users' information privacy concerns and the role of app permission requests. International Journal of Information Management, 50, pp. 261-272. https://doi.org/10.1016/j.ijinfomgt.2019.05.010

Gu, J., Xu, Y., Xu, H., Zhang, C., Ling, H. 2017. Privacy concerns for mobile app download: An elaboration likelihood model perspective. Decision Support Systems, 94, pp. 19-28. https://doi.org/10.1016/j.dss.2016.10.002

Wang, T., Duong, T. D., Chen, C. C. 2016. Intention to disclose personal information via mobile applications: A privacy calculus perspective. International Journal of Information Management, 36, pp. 531-542. https://doi.org/10.1016/j.ijinfomgt.2016.03.003

Xu, H., Teo, H.-H., Tan, B. C. Y., Agarwal, R. 2009. The Role of Push-Pull Technology in Privacy Calculus: The Case of Location-Based Services. Journal of Management Information Systems, 26(3), pp. 135-173. https://doi.org/10.2753/MIS0742-1222260305

Xu, H., Teo, H.-H., Tan, B. C. Y., Agarwal, R. 2012. Effects of Individual Self-Protection, Industry Self-Regulation, and Government Regulation on Privacy Concerns: A Study of Location-Based Services. Information Systems Research, 23(4), pp. 1342-1363. https://doi.org/10.1287/isre.1120.0416

How does your paper extend the current body of knowledge?

We know of no other work that has examined the public's attitudes towards different tracking technologies in such depth. We are not aware of any work addressing the public's attitudes towards immunity passports. We also are not aware of any work that has addressed the way in which perceived risk of a disease trades off against perceived harm of a remedial technology to determine people's acceptance of a privacy-encroaching technology. We suggest that we provide answers to all those questions, thereby adding to the current body of knowledge.

Minor issues:
- There are issues with the structure. Please start with an introduction and summarise the sections "Tracking technologies" and "Immunity passports" under, e.g., "Background".
- Change "sonsiderable" to "considerable" on line 77 on p. 4

We have fixed the typo.

[…]

While revising your submission, please upload your figure files to the Preflight Analysis and Conversion Engine (PACE) digital

diagnostic tool, https://pacev2.apexcovantage.com/. PACE helps ensure that figures meet PLOS requirements. To use PACE, you must first register as a user. Registration is free. Then, login and navigate to the UPLOAD tab, where you will find detailed instructions on how to use the tool. If you encounter any issues or have any questions when using PACE, please email PLOS at figures@plos.org. Please note that Supporting Information files do not need this step.

The figures were found to be in order using the PACE tool.
